# News and Notes

**Published:** 1902-08

**Authors:** 


### *NEWS AND NOTES.*

(We are indebted to the *Medical Age* for many of these notes.)

---

THE *Atlanta Medical and Surgical Journal* and *Southern Medical Record* having been consolidated into THE ATLANTA JOURNAL-RECORD OF MEDICINE, exchanges will please be sent only to the latter. We are getting many duplicates.

---

YELLOW FEVER has been epidemic for several months in Vera Cruz.

---

OVER $10,000 has been subscribed for the building of a hospital for incurables at Indianapolis.

---

THE course of instruction for the medical officers of the Canadian militia has been instituted at Ottawa.

---

DR. J. C. BATES of Norfolk, Va., was shot and killed on July 10 by a negro patient who was delirious.

---

AT the closing session of the American Medical Association at Saratoga it was decided to meet in New Orleans in May, 1903.

---

DR. W. OSLER, of Boston, has been honored by Trinity University of Toronto with the honorary degree of Doctor of Civil Law.

---

THE Hawaiian Board of Health is about to begin a series of experiments to determine the value of X-rays in the treatment of leprosy.

OUT of 129 candidates for appointment as assistant surgeon in the army, only eighteen passed the examinations which were recently held.

---

THE Governor of Maryland has appointed the following commission on tuberculosis: Prof. William Osler, Lilian Welch, and W. F. Hines.

---

WOMEN are to be admitted in future to Rush Medical College on the same footing with men. This will provide for more of the fair left-overs of the closed Northwestern.

---

IN various German schools courses of treatment for the cure of stuttering children have been instituted. It is estimated that 1½ per cent. of the children attending German schools stutter.

---

NEW YORK CITY is preparing to build a very large floating bathhouse at the foot of East Twenty-third street. It will cost $200,000, and will furnish accommodations for 18,000 people.

---

DR. OTIS FREEMAN, who it is claimed was the oldest practicing physician in America, died at Freehold, N. J., April 8. Dr. Freeman was born in New Hampshire on December 30, 1809.

---

ACCORDING to *American Medicine*, a woman physician, Dr. Conard, has resigned from the resident staff of the West Philadelphia Hospital because she had to go out late at night without an escort.

---

CHOLERA continues to spread in Manila. Among the American troops ninety deaths have occurred. The gravity of the epidemic in the island may be known from the figures—8899 cases and 6676 deaths.

JANE TOPPAN, the nurse of Lowell, Mass., has confessed to the killing, by poison, of thirty-one patients whom she had nursed. She has been pronounced insane, and has been sent to the asylum for life.

---

FROM the experiments which have been conducted by Dr. Reid Hunt, of Johns Hopkins Hospital, he concludes that wood alcohol, which has been the cause of so much blindness in the last two years, is itself a poison, and its poisonous properties are not due to the impurities present in it, as is often claimed.

---

THE grand jury of Chicago has reversed the finding of the coroner's jury in the case of the fire at St. Luke's Hospital, which destroyed the lives of twelve people. The coroner's jury found the hospital authorities negligent. The grand jury finds that everything possible was done to save the lives of the patients.

---

DR. BENJAMIN LEE, secretary of the State Board of Health of Pennsylvania, is quoted as saying that two weeks of hot weather will kill the germs of smallpox. They had weather of that sort at St. Pierre some weeks ago, but we hope that no such intemperate temperature is anticipated in any part of the United States.

---

THE United States Senate has agreed to an amendment to the general deficiency bill which provides for an appropiation of $45,-000 to cover all unpaid expenses incurred during the last illness of President McKinley at Buffalo, including compensation for the physicians in attendance. The bill provides that no part of the sum appropriated shall be paid to any person in the employ of the government. Under this provision neither Surgeon-General Rixey of the navy nor Dr. Eugene Wasdin of the U. S. Marine Hospital Service will receive any extra compensation for their services.

GEN. WILLIAM H. FORWOOD has succeeded General Sternberg as surgeon-general of the army. General Forwood was appointed assistant surgeon in the army in August, 1861. His services have been long and honorable, including the Civil War, Indian campaigns, the war with Spain, the insurrection in the Philippines, and the expedition in China. He was twice brevetted in the Civil War for faithful and meritorious services. His tenure of office will be short, as he will retire for age in September next.

---

AT the meeting of the American Congress of Tuberculosis, which was held in New York June 4 and 5, the following officers were elected for the ensuing year: Honorary president, Dr. Henry D. Holton, of Brattleboro, Vt.; president, Dr. Daniel Lewis, of New York; vice-presidents, Dr. J. A. Eagen of Illinois, Dr. Frank Paschal of San Antonio, Texas, Dr. E. J. Barrack of Toronto, Canada, Dr. I. A. Watson of Concord, N. H., Dr. Romola of Guatemala; secretary, Dr. George Brown, of Atlanta, Ga.; treasurer, Dr. P. H. Bryce, of Toronto, Canada.

---

IN the course of an article on food adulteration, Dr. de Lavarenne, editor of *La Presse Médicale*, refers to the number of things in which the use of saccharin is being extended. It is not only used to sweeten beer, but it is now also employed in the manufacture of syrups, jams, lemonades, wines (especially champagne), cider, brandy, pastry and chocolates. Special substances of this nature are on the market for sweetening cider and brandy. Among these sucramine may be mentioned, which is said to be seven hundred times sweeter than cane-sugar. Other products of the same kind are sugar extract (made in Switzerland), cannabin, etc. All these names are misleading, for the substances are only sugars in name, being coal-tar derivations. They are not foods. Moreover, their long-continued use may gravely affect the digestive functions. Dulcin, another sweetening body, which has been used as a substitute for saccharin, was given to a dog at the rate of one grain a day. The animal died in three weeks.—*American Medicine.*

A COMPLIMENTARY dinner was given to Dr. George M. Sternberg, the retiring surgeon-general of the United States Army, at Delmonico's on the evening of June 13, to give the physicians and surgeons an opportunity of expressing their appreciation of the work which that officer had done during his term of office. About one hundred well-known physicians and surgeons from various parts of the country were present, several of them making speeches explaining the progress which has been made toward alleviating the sufferings of sick or wounded soldiers during Dr. Sternberg's incumbency of the office of surgeon-general. The banquet was presided over by Dr. E. G. Janeway, of New York. Among those present were P. M. Rixey, surgeon-general of the U. S. Navy; Dr. William Osler, of Baltimore; Dr. Abraham Jacobi and Dr. Frank Billings, of Chicago; Assistant Surgeon-General Henry Lippencott, U. S. A.; Dr. J. D. Bryant; Assistant Surgeon-General A. H. Smith, U. S. A.; Dr. Robert F. Weir, Dr. I. N. Love, and Dr. Herman M. Briggs.—*New York Medical Journal.*

---

DR. SABOURIN, a well known French physician, has recently published in the *Presse Médicale* the result of the treatment of consumption in the French sanatorium of Durtol. Out of a total of 250 cases, Dr. Sabourin has analyzed the number of patients cured. By this is meant that for three months there have been neither expectoration nor cough, that the patient looks well, and shows no reaction after exercise or any other causes which produce a change in a tuberculous patient. On their arrival at the sanatorium examination showed that, out of the total of 250 cases, 100 had lesions which prevented all chance of a cure. Out of the 150 that were not too far gone 94 were discharged cured, which makes a percentage of sixty. Of the 100 who were incurable 2 did ultimately recover. Out of the 94 cured 10 have since had a relapse. There were, therefore, 84 patients who recovered out of a grand total of 250, which makes a percentage of thirty-four. The 94 patients were affected in the following manner: Fourteen had lesions of the first degree, 56 of the second, and 24 of the third degree.—*Journal of the American Medical Association.*

SENATOR HOAR, of Massachusetts, recently introduced an amendment to the sundry civil expense bill which was referred to the Committee on Education and Labor. This amendment provides for the establishment, in the Department of the Interior, of a laboratory for the study of the abnormal classes, such study being a development of work already begun under the Federal government. This work includes not only laboratory investigations, but also the collection of sociological and pathological data, especially as found in institutions for the criminal, pauper and defective classes; as observed in hospitals, schools and other institutions; also investigation of anarchistic criminals, mob influence, and like phenomena. The causes of social evils are to be sought out, with a view to lessening or preventing them. These results will be collected and published. The amendment also provides for a director in the laboratory at an annual salary of $3,500, to have the power to employ specialists to assist him, and such other help as may be necessary. For the expenses thus involved, together with the equipment of the laboratory, $410,000 is to be appropriated, to be available on the passage of the act.

---

THE Fourteenth International Congress of Medicine will be held in Madrid, Spain, from April 23d to 30th, 1903, under the patronage of their Majesties, the King of Spain and the Queen-Mother. The President of the Congress is Professor Julian Calle-jay Sanchez, the General Secretary is Dr. Angel Fernandez-Caro, and the General Treasurer is Professor José Gómez Ocaña. The preliminary statement of regulations and program has just been issued, and it announces that members of the Congress will be physicians, pharmacists, dentists, veterinary surgeons, and other persons working at branches of medical science, both Spaniards and foreigners, who have entered their names and paid their subscriptions. Other persons who possess scientific or professional titles, and who wish to take part in the work of the Congress, may share in it under the above conditions. The subscription is thirty pesetas, and this sum must be paid before the opening of the Congress to the General Secretary, Faculty of Medicine, Madrid. A card of membership will be sent to the subscriber. Until March 20th, 1903, subscriptions may be paid to the Secretary of the National Committee of the subscriber, but after that date subscriptions must be paid directly to the General Secretary at Madrid. Members will receive a summary of the proceedings of the Congress, and a full report of the work of the particular section which they join. The official languages of the Congress will be Spanish, French, English, German and Italian. Papers must be sent to the General Secretary before January 1st, 1903, to be certain of a place in the order of business. Papers presented later will be considered after the discussion of those regularly announced. Communications should be accompanied by a short abstract, which will be printed and distributed among the members of the Congress.—J. H. Huddleston, Secretary, American Committee, 126 West 85th St., New York City.

---

M. Neymarck, according to the New York *Sun*, has lately examined various economic, financial, and social causes that influence the birth-rate. Some of his results are summarized in what follows: He believes, in the first place, that the birth-rate will always diminish with the increase of "civilization," with "progress," in a country. In Germany the birth-rate was 42 per 1000 in 1875; in 1895 it was 36. In England the rate diminished from 36 to 29 in the same period. In France it fell from 26 to 25.2. The rate diminution is, therefore, least in France. Some of the economical causes influencing the birth-rate are: First, the increased cost of living, or, more accurately, the increased scale of comfort. As a matter of fact, it is not certain that the cost of life has increased, but our requirements have become greater. Secondly, the desire to insure increased comfort for oneself and one's family. "The father of a family limits his offspring in proportion to his egoism." Thirdly, the desire to establish one's children well in life is proved by curious statistics. In France there were 281,353 heritages in the direct line to divide 3469 millions of francs; of these 170,819 heritages, amounting to 2131

millions, were allotted to one or two children; 75,961, amounting to 926 millions, were divided between three or four children; and 34,673, amounting to 412 millions, were divided between five or more children. Fourthly, the reduction of the rate of interests runs parallel to the decrease in birth-rate. In France the birth-rate was, in 1872, 27.8 per 1000; in 1880, 25.6; in 1890, 22.9; in 1900, 22.4. The 3 per cents produced, in 1871, about 5½ per cent. on the investment. Fifthly, the increase of taxes and the indirect effect of the obligations of military service must also be considered. Sixthly, the entrance of women into competition with men as wage-earners. In France there are now 3,353,831 women who are thinking less of maternity, as they are more or less interested in their professions or trades. There are 3,861,599 single women, 1,808,838 families without children, 3,000,000 divorced or widowed persons without children—nearly 6,000,000 persons in all these categories.

---

THE Royal College of Physicians of London and the Royal College of surgeons of England have conjointly drawn up a detailed scheme for systematic investigation of cancer, and the next step will be an appeal to the public for a fund to provide, extend, equip, and maintain laboratories to be devoted exclusively to cancer research; to encourage researches on the subject of cancer within the United Kingdom or in the British Dominions beyond the seas; to assist in the development of cancer research departments in various hospitals and institutions approved by the executive Committee; and generally to provide means for systematic investigation in various other directions into the causes, prevention, and treatment of cancer. Should the object of the fund be attained by the discovery of the cause and nature of cancer and of an effective method of treatment, the Royal Colleges, with the consent of the trustees, shall be empowered to utilize the fund either (*a*) for equipping with the necessities for such treatment such hospitals as they may select, or (*b*) for forwarding research into other diseases. The fund shall, says *American Medicine*, be administered by a president, vice-president, five trustees, three of whom may be nominated by the donors of sums of £1,000 and upwards, and one each by the Royal Colleges of Physicians and Surgeons' honorary treasurer, general committee, and executive committee. The general committee includes all the officers already enumerated, the members of the executive committee, and also has one representative each from the Royal Colleges of Physicians and Surgeons, of Edinburgh and Ireland, from the Faculties of Physicians and Surgeons in Glasgow and the Royal Veterinary Colleges of London, Edinburgh, and Ireland, and from donors of sums of £1,000 and upward. The general committee shall meet from time to time to hear reports from the executive committee on the general progress of the scheme, account of expenditure, and the audited financial statement. The executive committee—consisting of (*a*) the president (for the time being) and two fellows of the Royal College of Physicians of London; (*b*) the president (for the time being) and two members of the Council of the Royal College of Surgeons of England; (*c*) two members of the laboratories committee of the Royal College of Physicians and Surgeons; (*d*) two members nominated by the general committee; (*e*) one member nominated by the Royal Society; (*f*) one member nominated by the Royal Veterinary College, London—shall have control of the income of the fund and shall be charged with the general supervision and management of the arrangements to carry out the objects of the fund; shall appoint the working and consultative staffs and draw up the regulations under which the work shall be conducted. The working staff shall consist of: (*a*) A general superintendent of the investigation who may be the director of the central laboratory; (*b*) assistants to the general superintendent and director; (*c*) any other persons who may be appointed to make special investigations. The consultative staff shall consist of persons skilled in scientific investigations, representatives of home and colonial government departments, physicians and surgeons attached to hospitals, statistical experts, and others appointed by the executive committee. The consultative staff may receive fees for attendance at meetings, and may be remunerated for any services performed in connection with the objects of the fund, on such conditions as the executive committee may determine.

